# Arabic gum/chitosan/Zn–NPs composite film maintains the quality of Hass avocado fruit by delaying ripening and activating enzymatic defense mechanisms

**DOI:** 10.1038/s41598-023-50642-y

**Published:** 2024-01-03

**Authors:** Ahmed A. Rashedy, Mahmoud E. Abd El-Aziz, Ahmed S. E. Abd-Allah, Hamed H. Hamed, Hala E. Emam, Eman A. A. Abd El-Moniem

**Affiliations:** 1 Pomology Department, Faculty of Agriculture, Cairo University, P.O. 12613, Giza, Egypt; 2 Polymers and Pigments Department, National Research Centre, 33 El Bohouth St., Dokki, P.O. 12622, Giza, Egypt; 3 Horticulture Crops Technology Department, National Research Centre, 33 El Bohouth St., Dokki, P.O. 12622, Giza, Egypt

**Keywords:** Biochemistry, Physiology, Plant sciences

## Abstract

Avocado fruit is a climacteric fruit that has a short life after harvest. Chitosan (Ch) and Arabic gum (AG) have a pronounced effect on the storability of fruits. This investigation aimed to determine the effect of individual or combined use of Ch and AG as well as Ch/AG enriched with 2, 4, 8% Zn–NPs on physio-biochemical attributes and antioxidant capacity of Hass avocado fruit during cold storage (7 °C). The result showed that Ch or AG alone succeeded in maintaining fruit quality of Hass fruit during cold storage. Also, combined application of Ch/AG was more effective than individual application of Ch or AG in reducing fruit weight and polyphenol oxidase activity (PPO) as well as increasing total antioxidant capacity (TAC) and malondialdehyde (MDA). Moreover, Ch/AG coating enriched with 8% Zn–NPs recorded the lowest fruit weight loss, fruit decay %, TSS fruit content, fruit firmness and improved fruit skin and pulp color significantly compared to Ch/AG and control. Coating with Ch/AG/2%Zn NPs recorded the highest peroxidase (POD) activity, while Ch/AG/8% Zn–NPs recorded the highest TAC and the lowest PPO activity. Moreover, enriched Ch/GA with Zn–NPs recorded the highest CAT and POD activity compared to the control. This study shows the efficiency of Ch/AG enriched with Zn–NPs on preserving Hass avocado fruit quality during cold storage by delaying ripening process and activating enzymatic defense mechanisms.

## Introduction

Avocado (*Persea americana* Mill) is a high-calorie fruit due to its high content of monounsaturated and polyunsaturated fatty acids. Additionally, it has a high nutritional value due to its content of nutrients (K, Mg, Fe, P), vitamins (B, C, E), and antioxidants^1–3^. In addition to the greater economic importance of international trade. It is considered a productive plants^4,5^. Hass avocados are among the world's most popular cultivars in retail and consumption, due to their organoleptic characteristics (Taste, appearance, smell, flavor and texture) and high nutritional value^1^. Avocado is classified as a climacteric fruit, which is highly perishable due to its high rate of fruit respiration and increased production of ethylene which leads to accelerated fruit ripening and reduced post-harvest life^6^. The large amounts of post-harvest fresh fruit losses are due to significant weight loss and shrinkage^7^. Another negative impact on the environment is caused by postharvest losses, which emit 4.2 tons of CO_2_ per ton of wasted food^8,9^. One method that has been developed to increase the storability of fruits is the use of edible coatings.

Chitosan (Ch) is a high molecular weight polysaccharide, serving as promising semi-permeable edible coatings substance due to its safe, biodegradable, biocompatible properties, antimicrobial activities and modulating fruit atmosphere as well reducing fruit weight loss resulting from internal moisture loss^10,11^. Previous research has shown that Ch coating improves fruit quality and storability of many perishable fruits, such as papayas^12^, litchis^10^, loquat^13^ and Red Raspberries^14^. However, Ch still has weak mechanical strength^15^. Thus, to enhance the properties of chitosan film, composite nano-coatings could be a promising technology. It has recently been shown that coatings containing nano-titanium dioxide (TiO_2_) and Ag/TiO_2_ nanocomposite help in prolonging postharvest life of strawberries and melons fruits^16–18^.

Arabic Gum (AG) or Gum Arabic is a dried gummy substance from *Acacia senegal* and related Acacia species^19^. It is considered one of the oldest and most popular remedies and has a therapeutic for chronic kidney diseases since 5000 years^20^. It has excellent water solubility, indigestibility and lower solution viscosity than the other gums^19^. Recent studies indicate its cardioprotective, renal, gut-protective, anticoagulant, antimicrobial and anti-inflammatory implications. It has achieved measurable success when used in drug, and nano-technology^21^. In tomato fruit, combined application of 6% CaCl_2_ and 10% gum Arabic reduced fruit weight loss, and decay percent of fruits as well as maintained fruit firmness, TA, and TSS were preserved compared with the control^22^**.** Coating mangoes by 10% AG and 1% Ch reduced decay incidence, weight loss, ethylene production and respiration rate^23^. Nowadays, several studies have used AG combined with Ch as an edible coating for extending shelf life of many fruit species such as mango^24^.

Zinc oxide nanoparticles (Zn–NPs) have multifunctional properties in inhibiting microbial growth^25–30^, cosmetics, drug delivery and medical devices^31^. Furthermore, the U.S. Food and Drug Administration has listed Zn–NPs as generally safe (21CFR182.8991; ^32^. The application of 0.5% Zn–NPs reduced weight loss and microbial load as well as maintained strawberry fruit with higher firmness, antioxidant capacity and vitamin C content ^33^. Moreover, Zn–NPs have been used as an antibacterial agent and packaging film additive^26,34^. However, there is not enough information available about direct application as coating for avocado fruit.

However, the individual or combined application of Ch and AG as well as AG/Ch + Zn–NPs as composite coatings and their physio-biochemical mechanisms for preserving perishable avocado fruits has not been studied. Therefore, this study aimed to was to evaluate the efficiency of Ch, AG, AG/Ch and AG/Ch enriched with Zn–NPs as coatings on Hass avocado fruit quality and its physio-biochemical mechanisms during cold storage.

## Material and methods

### Materials

Chitosan (Ch, medium molecular weight, De-acetylation ≈ 75%), polyvinyl alcohol (PVA; Mwt = 30,000), and zinc acetate were purchased from Sigma Aldrich. Acetic acid (AcOH) and glycerol were obtained from Elnasr Pharmaceutical Chemicals Co. All chemicals were used in analysis grade without any purification required. Permission was obtained to use the avocado variety in this study which complies with relevant institutional, national and international guidelines and legislation.

### Preparation of Arabic Gum (AG)/Chitosan (Ch)/Zn–NPs nanocomposite film

Nano Zinc oxide (Zn–NPs) was prepared by refluxing zinc acetate (3.942 g) in alkaline ethanol (1.44 g NaOH/1L ethanol) at 70°C for 2 h, after that deionized water was added. The Zn–NPs were obtained as fine white powder by centrifugation of the previous solution for 10 min at 5000 rpm, and then calcined for 2h at 500°C.

A 2% Ch solution was prepared by dissolving Ch in 1% acetic acid. AG solution (4%) was prepared by dissolving AG powder in distilled water at 40 °C for 1h using a magnetic stirrer. The composite AG/Ch was obtained by mixing Ch and AG solution by volume ratio 1:1 under a magnetic stirrer for 10 min. Finally, the nanocomposites were fabricated by the addition of various concentration of Zn–NPs to previous mixture to prepare 2, 4 and 8% AG/Ch/ZnO–NPs Nano composite. In addition, the glycerol was added by 10%/volume to all the prepared coating as plasticizers^35,36^. The composition of the as-prepared coatings was illustrated in Table 1.

**Table1:** Composition of prepared coating substances.

Substance	Ch 2% (ml)	GA 4% (ml)	ZnO–NPs (%)	WVT (g/m^2^.day)
AG/Ch	100	100	–	1060
AG/Ch/2% Zn–NPs	100	100	2	1421
AG/Ch/4% Zn–NPs	100	100	4	1191
AG/Ch/8% Zn–NPs	100	100	8	851
Ch	100	–	–	1862
AG	–	100	–	2675

### Characterization of coating materials

At room temperature, Fourier-transform infrared (FTIR) spectra between 400 and 4000 cm^−1^ were captured for as-prepared samples using a Mattson 5000 FT-IR spectrophotometer (Unicam, UK).

With a magnification of 600,000, a resolution power of 0.4 nm, and an operating voltage of 120 kV, the Jem 1230 Transmission Electron Microscope (TEM) from JEOL, Japan, was used to examine transmission electron micrographs. Scanning electron images were obtained by Quanta FEG-250 Scanning Electron the surface morphology of as-prepared samples was obtained by Quanta FEG-250 Scanning Electron Microscopy (SEM) instrument, Ametek Holland.

At room temperature, X-ray powder diffraction (XRD) patterns were captured using a Ni-filter and Cuk radiation source (*λ* = 1.54 Ǻ) operated at 40 kV and 40 mA in the 2*θ* range 5–80° at the scan speed of 0.05° per second on a Philips PW 1390 Diffractometer from Japan.

Under a nitrogen atmosphere, a Shimadzu TGA-50 thermo-gravimetric analyzer, Columbia, EUA, was used to carry out the thermal analysis in the range of 25–600 °C at a heating rate of 10 °C/min.

The cup method was used to measure the water vapour transmission rate (WVT, GBPI Co., China) using the GBPI W303-B Water Vapor Permeability Analyzer. WVT was determined as the volume of water vapour that permeates a given area during a given period at a given humidity level (4–10%) and temperature (38 °C). JIS Z0208, 53,122–1, TAPPI T464, ASTM D1653, ISO 2528, and ASTM E96 were followed in the assessments^35,36^.

### Fruit sampling

This study was conducted during 2021 at the National Research Center (Horticultural Crops Technology Lab and Department of Polymers and Pigments) and Cairo University (Pomology department, Faculty of Agriculture), Giza, Egypt. During October, high quality Hass avocados fruit were harvested from Salmiya private orchard (30°41′42″ N and 30°23′16″ E, elevation 9 m) in El Behera Governorate, Egypt. Avocado fruits were harvested at 20% dry matter mainly^37^ with full green color, firm stage (40 kg/cm^3^), uniform size and weight (150 g) as well as free from mechanical defects. The harvested fruit were carefully packed into open display boxes and immediately transferred to the Lab. The fruits were distributed to seven postharvest treatments: Ch, AG, AG/Ch, AG/Ch/2%ZnO–NPs, AG/Ch/4%ZnO–NPs, AG/Ch/8%ZnO–NPs and control.

### Edible coatings application

A total of seven treatments i.e., control, AG, Ch, AG/Ch, AG/Ch/2% ZnO–NPs, AG/Ch/4% ZnO–NPs, and AG/Ch/8% ZnO–NPs were used in this study. The fruits were divided into 7 groups, each one containing 60 fruits which were dipped in the previously prepared coating for 5 min, then dried (25 °C/20 min), packed in perforated boxes, and stored up to 3 months (7 ± 1 °C and 90% RH). During cold storage, the following fruit quality measurements were recorded every 15 days.

### Fruit physical quality parameters

#### Weight loss %

A digital balance was used to determine fruit weight loss percent using the following equation:

$$\mathrm{Weight\, loss \%}= \frac{\mathrm{Initial\, weight}-\mathrm{ weight\, at\, specified \,storage \,time }}{\mathrm{Initial \,weight}}\times 100$$

#### The fruit decay %

The percentage of fruit decay was determined visually by the following equation:

$$\mathrm{Fruit\, decay \%}= \frac{\mathrm{Number \,of\, rotten\, fruits\, at\, specified \,storage \,time }}{\mathrm{Total \,number\, of\, fruit }}\times 100$$

Decayed fruits were classified as moldy spots visible on the fruit surface.

#### Fruit firmness (kg/cm^3^)

Fruit Firmness (kg/cm^3^) was measured on the two opposite sides of each unpeeled fruit sample using penetrometer (FT327, FACCHINI srl, Alfonsine, Italy) according to Abd El-Moniem et al.^38^.

#### Fruit peel and pulp color

Fruit skin and pulp color were measured every two weeks according to Mcguire^39^. For skin color, three fruits for each replicate were labeled on the equatorial region (four regions/fruit) and then color on the same labeled spot was recorded. While for pulp color measurement, three fruits for each replicate were divided into 4 slices and then color was measured on each slice. Fruit color was measured by the colorimetric method using a Minolta Chroma Meter CR-2000 (Chroma Meter; Konica Minolta Sensing, Inc., Osaka, Japan). Values were recorded as L^*^ (lightness = 100, black = 0), b^*^ (yellow–blue scale) and a^*^ (green--red).

### Fruit chemical quality parameters

#### Total titratable acidity % (TA)

Total titratable acidity in the extracted juice was determined and expressed as citric acid % by titration with NaOH (0.1 N) to a stable faint pink color as end point using 2 drops of phenolphthalein (1gm solved in 100 mL ethanol) as indicator. Titration was performed in 20 mL of final solution (20 g of fruit pulp mixed in a blender with 100 mL of distilled water) and repeated three times. TA determination performed from A*B equation

$${\text{A}}=\frac{(\mathrm{volume\, of \,titrate }\times \mathrm{normality\, of \,titrate }(0.1\mathrm{ N})}{(\mathrm{volume\, of\, sample}\times 1000)}$$

$${\text{B}}=\frac{(\mathrm{Equivalent\, weight \,of\, citric\, acid }(64.04) }{ (\mathrm{volume\, of\, sample })}\times 100$$

#### Vitamin C (mg g^−1^FW)

In the previous extracted juice vitamin C (ascorbic acid) was determined using 2,6 dichlorophenolindophenol titration method^40^.

#### Fruit TSS content

TSS was determined by a digital hand refractometer (PR32, Atago Palete ATago CO.LTD. Japan) in the previous extracted juice and expressed as Brix^12^.

### Respiration rate

The respiration rate was measured on three avocados per replicate using the closed system method. It was measured based on the amount of CO_2_ produced by the fruits and per unit of fresh weight. The fruits were carefully placed in sealed 2-L glass jars for 24 h under the same experimental conditions. The respiration rate was then measured using an O_2_/CO_2_ gas analyzer (Model 1450-Servomex 1400, Japan) from the jar headspace through the rubber septum and expressed as mL of CO_2_ kg^−1^ fruit/h^41^.

### Enzyme analysis

Extraction and determination of fruit enzymes were performed in 1.0 g of frozen fresh fruit tissue^42^.

#### Extraction

A sample of the fruit was taken each time the sample was taken and immediately kept at − 30 °C for enzymatic analysis. To extract the enzymes, the mixture was formed by potassium phosphate buffer (50 mM) at pH 7.0 containing 1% PVP, 1 mM PMSF, 0.2 mM EDTA (w/v), and 0.1% Triton X100 (v/v). Extraction solution (5mL) was added to avocado tissue (1g), grounded with liquid nitrogen and homogenized. After centrifugation (22,000 g for 10 min, 4 °C) the supernatant was ready for the enzymatic assay.

#### Soluble proteins

The microassay procedure from protein assay kit (Bio-Rad) was used. Preparing reaction mixture composite of 80 μL sample, 200 μL of dye reagent and 720 μL of distilled water. This mixture was vortexed (DLAB MX-S, DLAB Scientific Co., Ltd, China), incubated at room temperature (30 min) and absorbance read at 595 nm (Optizen POP UV/VIS spectrophotometer, Serial POP, KLAB, Republic of Korea) and then BSA stock solution used as standard curve for proteins calculation.

#### Total antioxidant capacity (TAC)

The antioxidant capacity was obtained by mixing the enzyme extract (20 µL) and 125 µL of 60 µM of 2,2-diphenyl-1-picrylhydrazyl (DPPH). This mixture was then incubated (30 min) in the dark and the absorbance was measured at 517 nm (Thermo Scientific Genesys, China). TAC was calculated using a trolox as a standard curve and was expressed as µmoles g^−1^ DW^43^.

#### Catalase activity (CAT)

The enzyme mixture included phosphate buffer (2.55 mL) at pH 7.0, 50 mM, H_2_O_2_ (250 μL) and enzyme extract (200 μL). The absorption reduction of H_2_O_2_ (37.5 mM) at 240 nm was used to determine CAT activity expressed in U mg^−1^ of protein..

#### Lipid peroxidation via malondialdehyde (MDA)

Lipid peroxidation via malondialdehyde (MDA) was determinded according to Bailly and Kranner^44^ protocol. Avocado tissue (0.5 g) was ground and homogenised with 0.5% thiobarbituric acid (5 mL) and 20% trichloroacetic acid. This mixture was heated at 95 °C (water bath) during 30 min, then rapidly cooled on ice. The mixture was then centrifuged (22000 g for 2 min) and the supernatant (2 mL) used to measure MDA absorbance (532 and 600 nm). Then MDA was calculated and expressed as U mg^−1^ of protein of fresh weight.

#### Peroxidase activity (POD)

Peroxidase activity (POD) was assessed by mixing the enzyme extract(140 μL) and 570 μL of guaiacol (20 mM). Then the mixture was incubated (5 min/30 °C) and then added 290 μL of H_2_O_2_ (50 mM) and the absorbance read (460 nm) every 30 s for 5 min and POD expressed in U mg^−1^ of protein.

#### Polyphenol oxidase activity (PPO)

Polyphenol oxidase activity (PPO) was assessed by mixing the enzyme extract (0.1 mL), grade water (0.9 mL), 0.001 M catechol (1 mL), 0.5 M K-phosphate buffer (1 mL) at pH 6.5. Then PPO activity was determined within 3 min by monitoring the increase of absorbance (280 nm) according to Worthington and Worthington^45^ and PPO activity was expressed in U mg^−1^ of protein.

#### Statistical analysis

The design of this experiment was in a two way factorial. The treatments layout in a randomized complete block design (RCBD) with two factors (storage periods and postharvest materials) for employ treatments with three replicates for each treatment. Analysis of variance (ANOVA) was calculated via MSTAT-C software package^46^. Treatments means were compared by the least significant difference test at level of *p* < 0.05^47^. The data presented in the figures are means ± standard error (SE).

### Ethics approval

The authors confirmed that the institutional committee and licensing committee approved the experiments, including all relevant details and that all experiments have been performed in accordance with specified named guidelines and regulations.

### Ethics approval and consent to participate

All methods were in accordance with relevant institutional, national, and international guidelines and legislation

## Results and discussion

### Characteristics of ZnO–NPs

**Figure 1:**
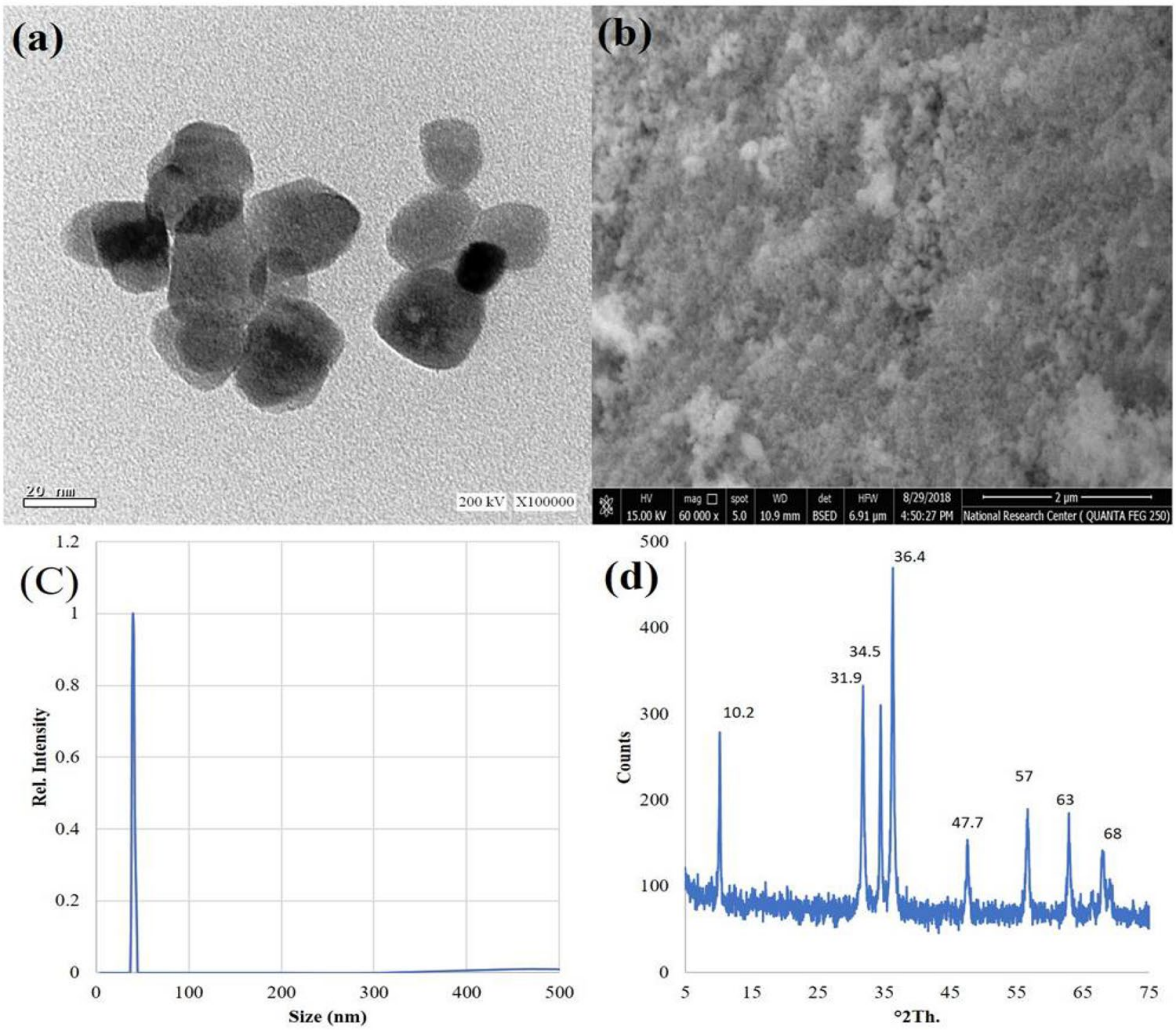
Transmission Electron Microscope TEM (**a**), Scanning Electron Microscopy SEM (**b**), particles size distribution (**C**), and XRD patterns of Zn–NPs (**d**).

The morphological characters of as-prepared ZnO–NPs were illustrated in Fig. 1a, b using TEM and SEM, respectively, which illustrated that ZnO–NPs have particles size less than 50 nm. This results were confirmed by using particles size distribution (Fig. 1c) which demonstrated that the particles size of ZnO–NPs has narrow size and less than 50 nm.

The crystal structure of the ZnO–NPs was represented in Fig. 1d, where the XRD partum showed the peaks at main peaks 2θ = 31.9, 34.5, 36.4, 47.7°, and 57° that are corresponding to ZnO–NPs which are related to plans (100), (002), (101), (102) and (110), respectively.

### Characterizations of coating films

**Figure 2:**
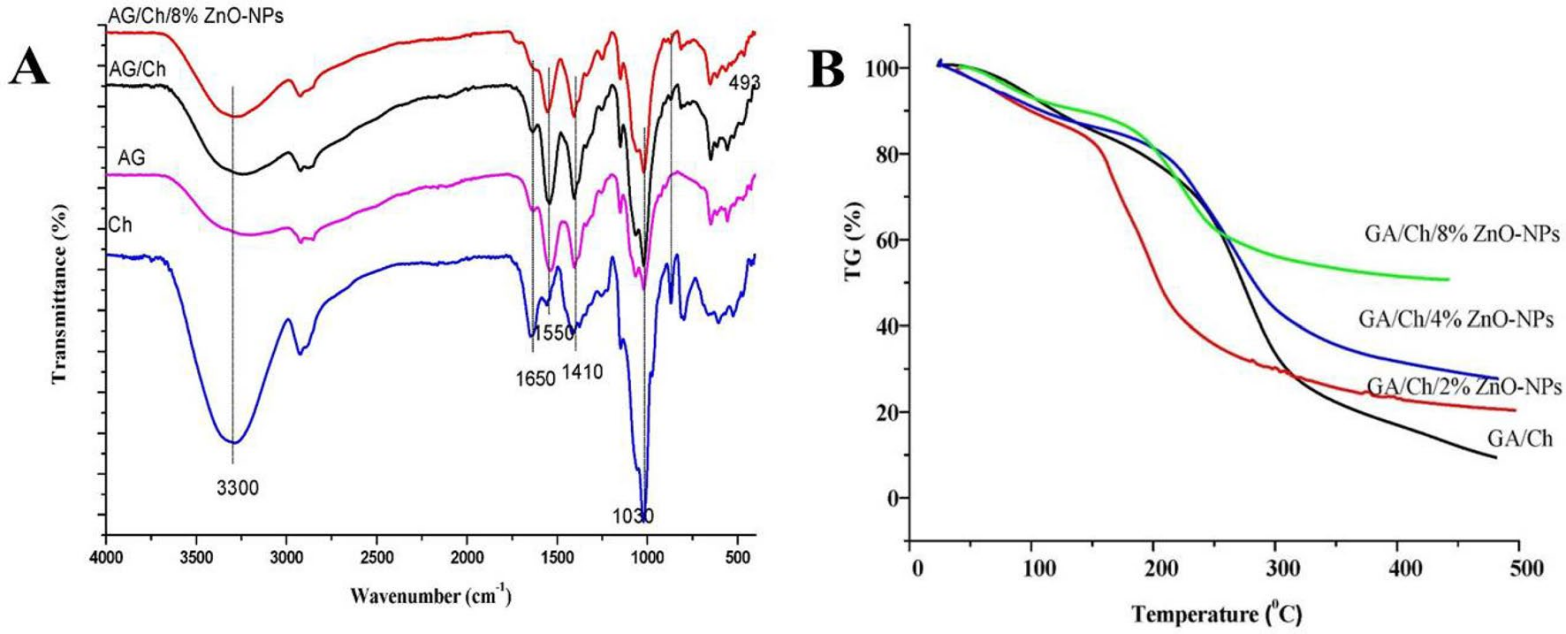
Fourier-transform infrared FTIR (**A**) and Thermogravimetric analysis TGA (**B**) of as-prepared coatings (chitosan (Ch), Arabic gum (AG), AG/Ch, and AG/Ch/8% Zn–NPs).

FT-IR spectrum of as-prepared coatings was obtained in Fig. 2A. All the prepared samples exhibited the absorption band at bands 3300, 1650, 1410, and 1030 cm^−1^ corresponding to –OH and –NH stretching vibration, C=O and C–O stretching of the amide group, and C–O stretching, respectively. These results were in consistent with Mahmoud et al.^48^. on green snap bean using of chitosan and chitosan nanoparticles. Also, Salama et al.^49^. on common bean (*Phaseolus vulgaris* L.) using nanofertilizer.

The coating GA/Ch/8% ZnO–NPs exhibited an additional band in the range of 500–1000 corresponding to inorganic materials at 493 cm^−1^ in response of Zn–O stretching vibration.

Thermogravimetric analysis (TGA) was carried out in nitrogen atmospheres to look into the thermal stability of the as-prepared coatings. According to Fig. 2B, the coating Ch/AG and its nanocomposites exhibited two steps of decomposition. The first step is evaporating of adhering and adsorbed water while the main degradation process during TGA analysis is due to the processes of polymer chain degradation through end group-initiated mechanism and the thermal degradation of the products formed during polymer chain degradation. while the as-prepared nanocomposite coatings showed relative stability over Ch/AG coating due to the presence of ZnO–NPs, and this stability grew when ZnO–NPs loading was increased.

Transmission of water vapour (WVT), which measures how easily moisture can be absorbed and distributed through a material, has a significant impact on how packing materials are prepared to affect food shelf life. The WVT data of as-prepared coated films were illustrated in the Table 1. The data showed that the inclusion of Ch improved the blend film’s (AG/Ch) moisture barrier over the pure GA, reducing WVTR for the created blend AG/Ch. In addition, increasing the loading of ZnO–NPs in nanocomposite films creates a significant decrease in water transmissibility, since the existence of ZnO–NPs promotes the hydrophobicity of nanocomposite films. These results were in agreement with Youssef et al.^36^. in preserving fresh chicken breast fillets by using TiO_2_-NPs and cinnamon Nanoemulsion.

### Fruit physical quality parameters

#### Fruit weight loss

**Figure 3:**
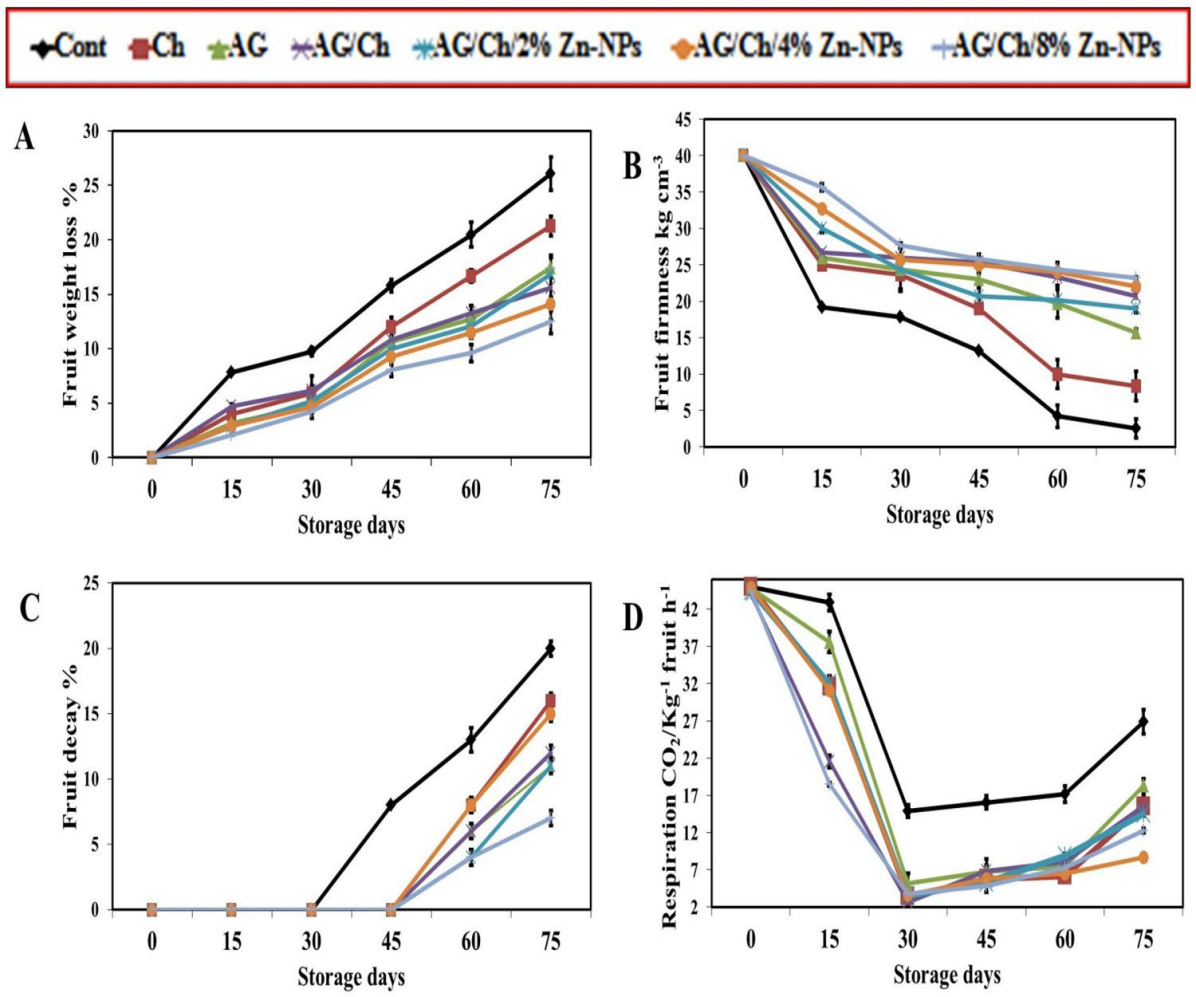
Effect of chitosan (Ch), Arabic gum (AG), AG/Ch, and AG/Ch enriched with 2, 4, 8% Zn–NPs on fruit weight loss (**A**), firmness (**B**), decay (**C**) and respiration rate (**D**) of Hass avocado fruit during cold storage (7C). Data were presented as means (n = 3 ± SE).

As shown in Fig. 3A, the percentage of weight loss for avocado samples increased with increasing storage periods, but the rate of weight loss for the control (Cont) treatment was significantly higher than of all coated treatments. At 75 days of cold storage, application of AG/Ch as composite coating delayed avocado fruit weight loss (15.62%) compared to Ch (21.27%), AG (17.46%) and control (26.07%). Moreover, at 75 days of cold storage, AG/Ch + 8% Zn–NPs composite treatment was the most effective treatment in reducing weight loss (12.5%) compared to control (26.07%) and all other treatments. These results were in line with Tahir et al.^50^ who found the reduction of strawberry weight loss by Arabic Gum as coating. In tomato fruit, combined application of 6% CaCl_2_ and 10% gum Arabic reduced fruit weight loss compared with the control fruit^22^. Also, Coating mangoes with mixture of AG (10%) and Ch (1%) reduced weight loss^23^. Also, AG 15% + Moringa and CMC (carboxy methyl cellulose)1% + M retained fruit firmness and lowered weight loss of Maluma avocado fruit^51^. Moreover, AG 15% + Moringa decreased weight loss of Maluma avocado fruit^51^. weight loss percent of Hass avocado increased significantly during cold storage, while beetroot and turmeric extract extract significantly decreased it.

The weight loss in fruit results from water from water evaporation due to the processes of respiration and transpiration^33^. Tahir et al.^50^. reported that 15% AG-coating for strawberry significantly reduced weight loss. Chitosan coating forms a semi-permeable layer that creates a modified atmosphere that protects against O_2_, CO_2_, H_2_O and water loss^52,53^. Also, AG is a polysaccharide with functional hydrophilic properties, which had mechanical strength and slows gas exchange and moisture loss^54,55^.

#### Fruit firmness

The firmness of the fruit was significantly affected during storage so that the firmness of the control treatment from the 15th day to the 75th day went from 19.20 to 2.53 kg/cm^3^ and the firmness of the chitosan treatment Enriched decreased from 35.67 to 23.17 kg/cm^3^ (Fig. 3B). AG had significantly higher fruit firmness than Ch at the different storage periods except for 15 days. Also, AG/Ch recorded the highest significant fruit firmness followed by Ch then control at 45, 60 and 75 days of storage. Moreover, AG/Ch + 8% Zn–NPs composite coating recorded the highest significant fruit firmness during 15, 30, 60 and 75 days of storage compared to all treatments. These results were in agreement with Kubheka et al.^51^ who found that AG(15%) + Moringa maintained high fruit firmness of Maluma avocado fruit^51^. Also, Coyotl-Pérez et al.^56^ reported high firmness of avocado fruit treated with Ch + essential Oil. The efficiency of AG on maintaining high fruit firmness during cold storage was recorded in many fruit species such as apple^57^ and mango^23^. In tomato fruit, combined application of 6% CaCl_2_ and 10% gum Arabic maintained fruit firmness compared with the control fruit^22^.

Tahir et al.^50^ reported that, 15% AG-coating for strawberry significantly improved fruit firmness. Furthermore, 0.5% Zn–NPs maintained higher firmness of strawberry fruit^33^. Softening of fruit resulting from cell structure deterioration in both intracellular materials and the cell wall^58^. Coating the fruit with AG/Ch maintains high fruit firmness by covering the lenticels and cuticle which reduces respiration and delays fruit ripening^59^. Arabic gum(10%) + 1000 ppm *Moringa oleifera* seed oil treatment maintain fruit firmness of Hass avocado fruit^38^.

#### Fruit decay%

The results indicated that the rate of decay in the fruits increased with long storage periods. Up to 45 days of cold storage, all coating treatments successfully prevented fruit decay (Fig. 3C). Moreover, both AG and AG/Ch-coated avocadoes significantly reduced fruit decay by 11% and 12% compared to Ch alone (16%), while control had the highest decay rate (20%) at 75 days of storage. The minimum percentage of rotting of avocados coated with gum arabic/chitosan was observed in concentrations of 2 and 8%, which showed a significant difference with other treatments, including the concentration of 4%. Moreover, at the end of the storage period, the treatment AG/Ch + 8% Zn–NPs recorded the lowest percentage of decay compared to other treatments. Therefore, AG/Chcoating treatment with Zn–NPs could be more effective in preventing fruit decay.

In this regard, Khaliq et al.^24^ reported that chitosan coatings on the fruit surface acts as a barrier that protects the fruits from infection by pathogen, thereby reducing fruit decay. Also, a combined coating of Arabic gum (10%) and chitosan (1%) reduced mango fruit decay^23^. Also, Ch reduced fruit decay of Red Raspberries ^14^. Moreover, GA-coating (15%) for strawberry significantly inhibited fungal infections^50^. Rai-Kalal et al.^60^ found a reduction in strawberry decay due to treatment of 0.5% Zn–NPs. Ch inhibits the development of decay-causing pathogens, and stimulates host tissue resistance response^61^. Also, Ch inhibits the development of decay-causing pathogens and stimulates host tissue resistance response^61^. Recently, in a study, the use of a combination of chitosan and titanium oxide nanoparticles reduced the percentage of decay in mango fruit. Weight loss percent of Hass avocado fruit increased gradually during cold storage^62^.

### Respiration

The respiration rate decreased suddenly from day 15–30, but from day 30 onwards we saw an increase in the respiration rate of fruits (Fig. 3D). At the end of storage period, AG (18.28 CO_2_kg^−1^fruit h^−1^), Ch (15.53 CO_2_kg^−1^fruit h^−1^) and AG/Ch (15.63 CO_2_kg^−1^fruit h^−1^) significantly reduced respiration rate compared to control (42.97 CO_2_kg^−1^fruit h^−1^). Also, AG/Ch + 4% Zn–NPs recorded the lowest respiration rate (8.70 CO_2_kg^−1^fruit h^−1^) compared to all treatments followed by AG/Ch + 8% Zn–NPs (12.27 CO_2_kg^−1^fruit h^−1^). In this regard, chitosan layer formed on the fruit surface modifies the gas and atmosphere exchange, directly affecting the respiration and ripening of the fruit^12^. Also, Handojo et al.^23^ reported that Arabic gum (10%) and chitosan (1%) reduced respiration rate and ethylene production of mango fruit^23^. Also, Khaliq et al.^24^ reported that coated mangoes using Arabic gum (10%) and chitosan (1%) reduced respiration rate and ethylene production. Moreover, chitosan/Nano-TiO_2_ composite decreased decay of mango fruits and the respiration rate peak appeared 5 days later^63^.

Respiration rate in avocado fruit gradually decreased during cold storage period up to 30 days of cold storage and then gradually increased in all treatments. Climacteric peak for all treatments cleared after 45 days of cold storage, with the value of each treatment varying, which led to a delay in fruit ripening. At the end of storage period (75 days) Ch/Ag + 4%Zn–NPs followed by Ch/Ag + 8%Zn–NPs and Ch/Ag + 2%Zn–NPs recorded the lowest respiration rate. In addition, individual coating of avocado fruit with Ch, GA and Ch/GA alone or enriched with Zn–NPs has the ability to form a mechanical barrier that helps control respiration rate control, prevent water loss, maintain high fruit firmness and extend storage period.

### Fruit chemical quality parameters:

#### Vitamin C content

**Figure 4:**
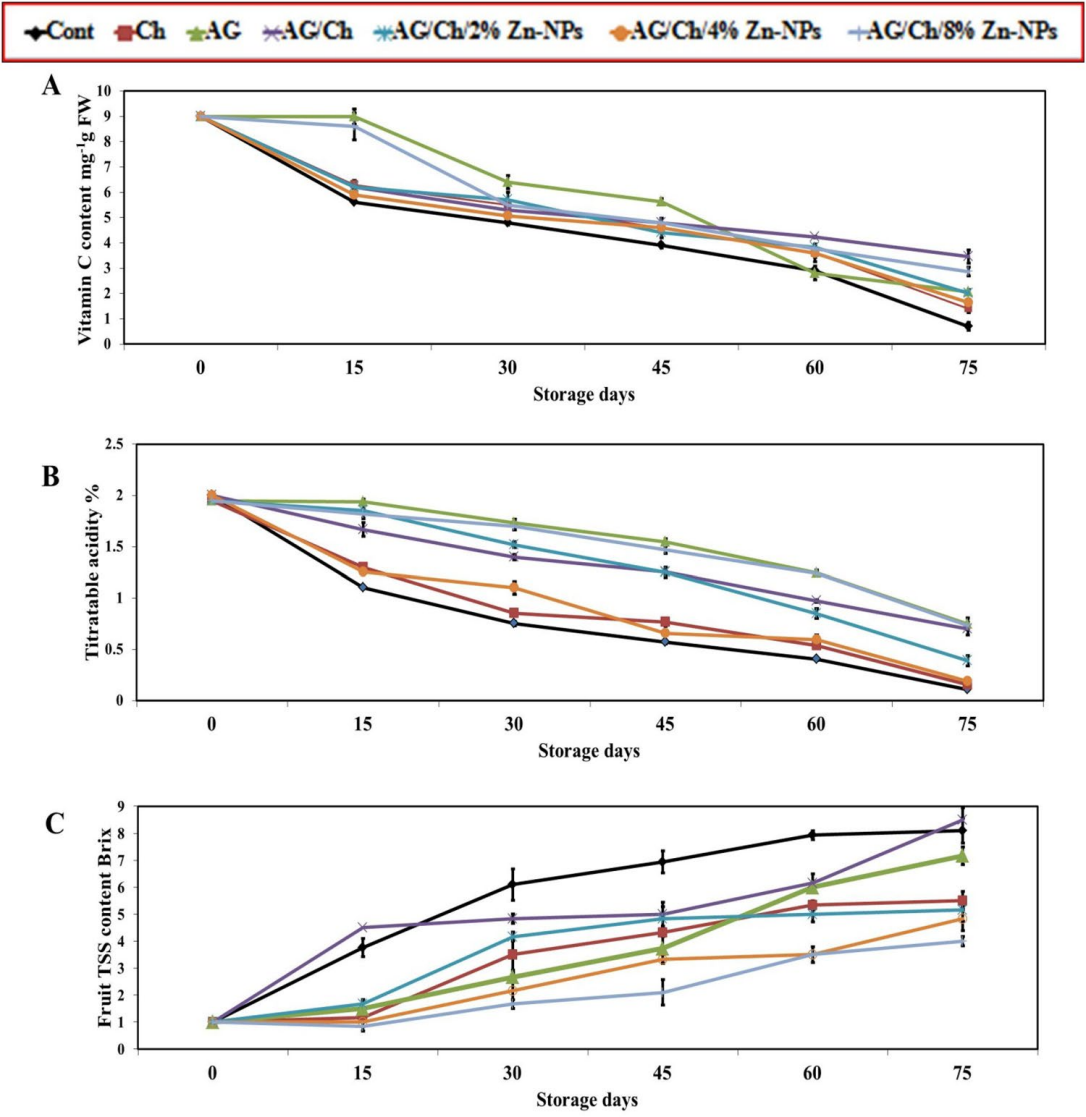
Effect of chitosan (Ch), Arabic gum (AG), AG/Ch, and AG/Ch enriched with 2, 4, 8% Zn–NPs on fruit Vitamin C content (**A**), titratable acidity (**B**), TSS content of Hass avocado fruit during cold storage (7C). Data were presented as means (n = 3 ± SE).

Vitamin C content decreased gradually with increasing storage periods from 15 till 75 days (Fig. 4A). At the end of storage period, treatments of AG/Ch (3.47 mg^−1^FW) followed by AG (2.07 mg^−1^FW) preserved higher significant vitamin C content compared to control (0.7 mg^−1^FW) and Ch (1.4 mg^−1^FW) alone. Moreover, AG/Ch + 8% Zn–NPs (2.86 mg^−1^FW) recorded higher vitamin C content compared to all treatments except for AG/Ch treatment. In this regard, vitamin C content significantly decreased during cold storage of “Hass” avocado fruit^62^. Also, Hong et al.^64^ reported that a 2% chitosan coating was more effective in delaying the reduction of vitamin C of guava fruit than 0.5% and 1%. Moreover, strawberry fruit treated with 0.5% Zn–NPs maintained a higher concentration of vitamin C than untreated fruit^33^. The decrease in Vitamin C with increasing storage periods may be due to auto-oxidation resulted from combination of ascorbic acid with oxygen in the air^33^. A decrease in vitamin C content has been reported in “Hass” avocado fruit during cold storage^38,62^. While AG enriched with moringa or algae extracts succeeded in preserving a high percentage of vitamin C in Hass avocado fruit ^38^.

#### Fruit titratable acidity

Avocado fruits recorded high titratable acidity during 15 days of cold storage, and then decreased gradually until the end of storage periods (Fig. 4B). Up to 60 days of storage, AG (1.2%) recorded a significant higher fruit acidity content than AG/Ch (0.97%), Ch (0.54%) and control (0.40%). Also, AG/Ch + 8% Zn–NPs (1.24%) and AG (1.24%) treatments recorded the highest significant titratable acidity in ‘Hass’ fruit at the end of storage. Citric acid is the main organic acid in avocado fruit. A decrease in titratable acidity during cold storage were recorded in many fruit species such as avocado^38,62^, strawberry^33^, and this may be due to the use of organic acids in the respiration process. Also, Hong et al.^64^ reported that a 2% chitosan coating was more effective in delaying the reduction of titratable acidity in guava fruit than 0.5% and 1% Ch which results from the ability of chitosan to modify the fruit internal atmosphere which preventing the reduction of titatable acidity.

#### Fruit TSS content

Figure 4C shows the changes in the TSS content of avocado during the cold storage periods. Fruit TSS content increased during storage period. Treatments of AG and Ch recorded lower TSS significantly compared to control but compared to Zn–NPs treatments, they showed more soluble solids on day 75. Moreover, composite treatments of AG/Ch + Zn–NPs at 4% and 8% recorded the lowest significant TSS at 75 days of storage compared to AG/Ch. In tomato fruit, combined application of 6% CaCl_2_ and 10% gum Arabic maintained fruit TSS compared with the control fruit^22^.

The reduction in fruit TSS content has been previously reported in chitosan-treated mangoes^12^. Also, Arabic Gum delayed the increase in TSS of Apples^57^ and strawberries^50^. Moreover, a composite of chitosan (1%) and Arabic gum (10%) change in TSS of mangoes^23^. Increasing TSS content during cold storage may due to the solubility of hemicelluloses and polyuronides which presented in cell wall^64^. Also, water loss due to transpiration might cause an increase in TSS^65^.

#### Fruit skin color

**Figure 5:**
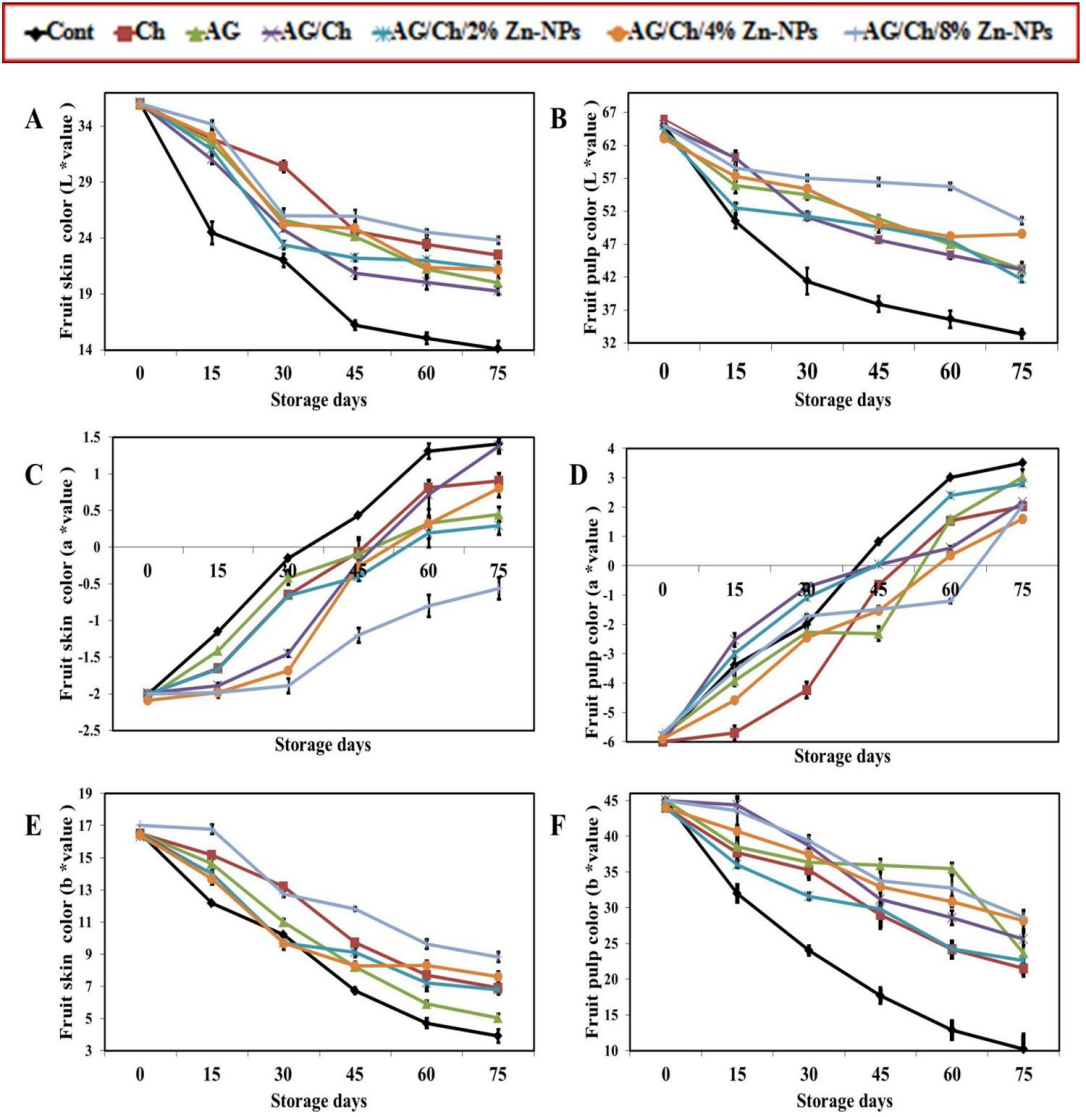
Effect of chitosan (Ch), Arabic gum (AG), AG/Ch, and AG/Ch enriched with 2, 4, 8% Zn–NPs on fruit skin color L^*^ value (**A**), a^*^ value (**C**), b^*^ value (**E**) and pulp color L^*^ value ^*^ (**B**), a^*^ value (**D**), b^*^ value (**F**) of Hass avocado fruit during cold storage (7C). Data were presented as means (n = 3 ± SE).

Fruit color is the primary and most widely used perception parameter in determining fresh horticultural produce quality. Changes in L^*^, a^*^, and b^*^ values of avocado skin color are shown in Fig. 5. For fruit skin color L^*^ gradually decreased with increasing storage periods (Fig. 5A). Combined (19.27) or individual application of AG (20.02) and Ch (22.50) maintained high significant fruit lightness compared to control (14.10) at 75 days of storage. Also, Ch alone recorded a significantly higher L value for fruit skin (30.40, 23.44, 22.50) than AG (25.62, 21.21, 20.02) and AG/Ch (24.75, 20.07, 19.27) through 30, 60 and 75 days of storage. Moreover, Zn–NPs treatments recorded a significant higher L-value compared to Cont. While, AG/Ch + 8% Zn–NPs succeeded in maintaining a significantly higher L-value (25.98, 24.52, 23.83) during 45, 60 and 75 days of storage period. With regard to fruit skin color a^*^ value it was increased with increasing storage periods (Fig. 5C). AG maintained lower a^*^ value (0.32, 0.28) during 60 and 75 days after cold storage than control (1.31, 1.41). Also, AG/Ch + 4% Zn–NPs significantly decreased a^*^ value (− 0.56) in fruit skin compared to control (1.41) at 75 days of storage. The highest values were recorded at 75 days of cold storage with AG/Ch (1.38) and control (1.41). Moreover, AG/Ch + 8% Zn–NPs gave the least significant values during all storage periods. Regarding fruit skin color (b value), the b-value of avocado skin color decreased gradually during storage periods (Fig. 5E). After 75 days of cold storage, Ch alone (6.91) significantly increased b-value during the storage periods compared to AG (5.04) and control (3.91) as did AG/Ch (6.80). Also, all Zn–NPs treatments increased color (b-value) significantly compared to control at 45, 60 and 75 days of storage. Moreover, AG/Ch + 8% Zn–NPs recorded the highest significant b-value during all storage periods except with Ch at 30 days. At 75 days of cold storage, AG/Ch + 8% Zn–NPs recorded the highest b-value (8.83).

The increase in purple (black) skin (lower b^*^ value) was predictable in dark-skinned avocados, as this refers to the anthocyanin synthesis and chlorophyll degradation, which coincided with avocado ripening^66^. This result confirms the findings of Adjouman et al.^67^ who reported delaying in a^*^ value increase in tomatoes due to polysaccharide coatings. The ability of these coatings to retain the green color of the avocado indicates their ability to delay the ripening of the fruits. Also, Jeong et al.^68^ and Cox et al.^66^ who reported delaying color change (green to dark purple) could extend dark-skinned cultivars shelf life. Conversely, the results showed there were sharp declines in b^*^ values in control than coated fruit during storage time. Maftoonazad et al.^69^ reported a decrease in b^*^ values in avocado indicating an increase towards darker chroma and decrease in yellowness. The color change was enhanced in the uncoated fruit and attained purple to black color during storage compared to the coated one. Although the fruit coated with AG 15% + Moringa and AG 10% + Moringa remained green up to 28 days of storage, this may result from coating materials (AG) providing a thick barrier against gas exchange and ethylene production between outer and inner environments, which delayed fruit ripening during storage.

#### Fruit pulp color L value

The L-value of fruit pulp (internal) color decreased from 60 at 15 days of cold storage to 33 at the end of cold storage period (Fig. 5B). Individual or combined use of Ch and AG resulted in a significant higher L-value in fruit pulp compared to Cont. Also, AG recorded a significant higher L-value (54.55, 50.88, 46.95) than Ch (51.10, 47.63, 45.33) and AG/Ch (51.10, 47.63, 45.33) at 30, 45 and 60 days of storage. During 15 days of cold storage AG/Ch had the highest significant L-values, while after 30, 45, 60 and 75 days of cold storage AG/Ch + 8% Zn–NPs had the highest significant L-values (57.06, 56.46, 55.81, 50.57) compared to all treatments. For fruit pulp color a^*^ value it was decreased from -5 at 15 days of cold storage to 0.2 at 75 days of cold storage (Fig. 5D). Generally, GA (− 2.32, 1.57, 3.03), Ch (− 0.67, 1.52, 2.03) and AG/Ch (0.04, 0.6, 2.16) maintained a less significant pulp a^*^ value compared to control (0.82, 3, 3.52) at 45, 60 and 75 days of storage. After 75 days of storage Ch (2.03), AG/Ch (2.16) or 2% (2.8) or 8% (2.07) AG/Ch + Zn–NPs recorded a significantly lower a^*^ value compared to all other treatments. Regarding fruit pulp color, b-value decreased gradually from 44 at 15 days of cold storage to 22 at 75 days of cold storage (Fig. 5F). Generally AG and Ch maintain a higher significant pulp b-value compared to Cont. Meanwhile, AG/Ch recorded the highest values (44.37, 38.67, 25.62) at 15, 30, 75 days of storage compared to Ch (37.74, 35.30, 21.54), GA (38.53, 36.37, 23.64) and control (26.44, 24, 10.24). A individual application of GA maintained a significantly higher pulp b-value (35.93, 35.44) higher than Ch (28.99, 24.15) during 45 and 60 days of cold storage. During 45, 60 and 75 days of cold storage Ch/AG enriched with 4% (32.96, 30.85, 28.20) and 8% (33.77, 32.72, 28.64) Zn–NPs recorded the highest significant fruit pulp b-value compared to control (17.69, 12.85, 10.24) and Ch (28.99, 24.15, 21.54).

After 28 days of storage, Maluma avocado fruit coated with Arabic Gum at 10% or 15% + Moringa recorded the highest pulp color scores. These results were consistent with Aguiló-Aguayo et al.^70^ who reported that, L^*^ values of fresh cut avocado showed a significant decrease in both untreated and pulsed light -treated samples after 15 days of storage, which indicated oxidative browning evidence during storage. Also, Gómez-López^71^ reported that Lightness (L^*^) is considered to be the most indicator for enzymatic browning in avocado fruit. Moreover, Light microscopy of apple fruit confirmed that color modifications were associated with cellular membranes breakage, which reduces the function of cell compartmentalization, and increase enzyme–substrate involved in tissue browning^70,72^. Severity of grey pulp in Avocado fruit significantly by MAP(modified atmosphere packaging) + Thyme oil treatment^73^. Gray pulp disorder occurs in cold-stored fruits during ripening^74^ which is associated with fruit senescence. Polyphenol oxidase (PPO) enzyme is responsible for this disorder in avocado^75^.

Color was recorded using the CIE L^*^a^*^b^*^ uniform color space (CIE Laboratories), where a* indicates chromaticity on a green (-) to red ( +) axis, L* indicates lightness and b* chromaticity on a blue (-) to yellow ( +) axis^76^. Higher color L (more lightness) and color b (more green) plus lower color a (more blue) means that the fruit was in more freshness and quality which was recorded in 8% Zn–NPs treatment compared to lower lightness (L) and red color (a) as well as a higher yellow color (b), which means that the fruit are more ripening, which is what was recorded in the control treatments.

### Enzyme analysis

**Figure 6:**
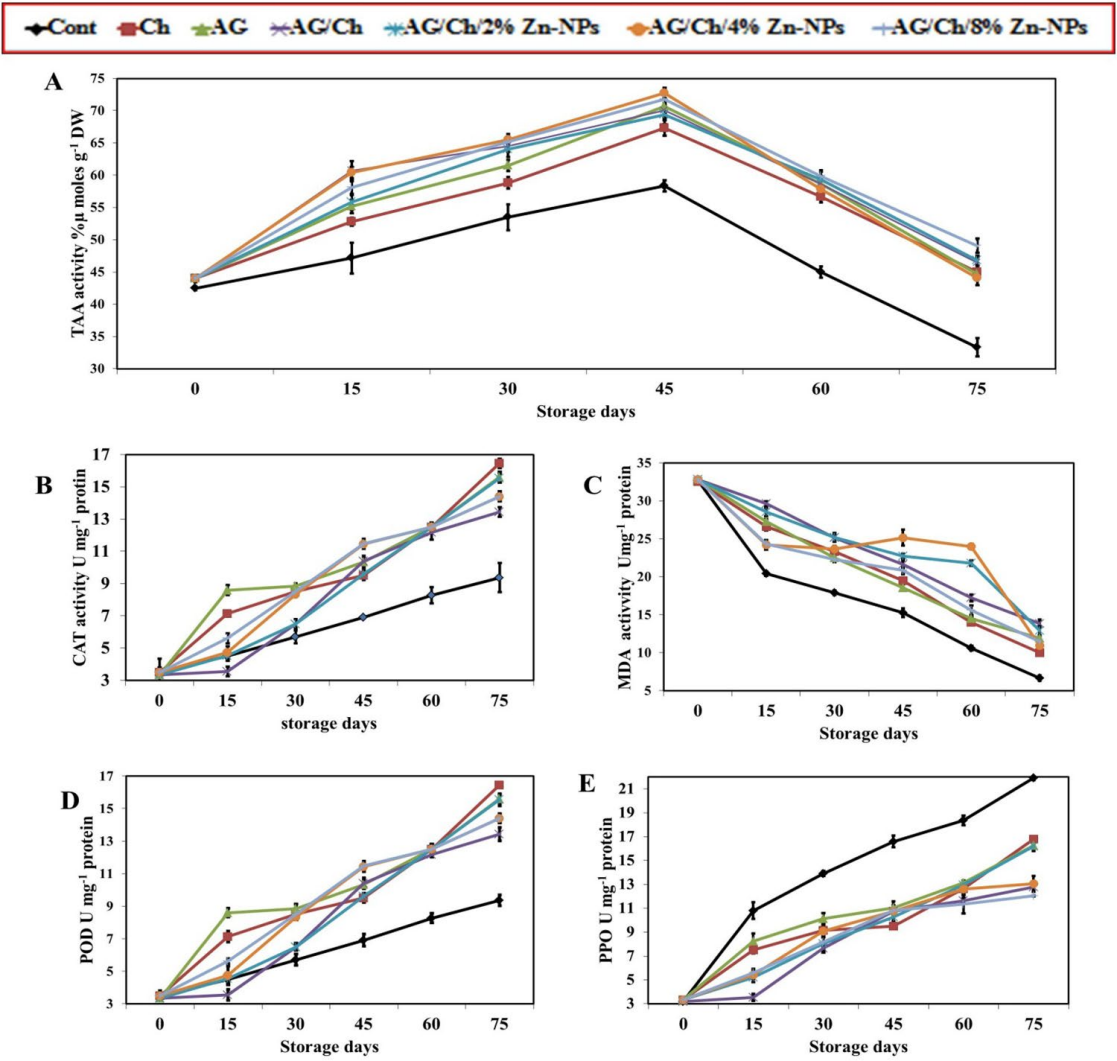
Effect of chitosan (Ch), Arabic gum (AG), AG/Ch, and AG/Ch enriched with 2, 4, 8% Zn–NPs on fruit Total antioxidant capacity TAC (**A**), catalase activity (CAT) (**B**), malondealdehyde (MDA) (**C**), peroxidase activity (POD (**D**), and polyphenoloxidase (PPO) (**E**) of Hass avocado fruit during cold storage (7C). Data were presented as means (n = 3 ± SE).

TAC gradually increased with increasing storage period up to 45 days of storage and then gradually decreased (Fig. 6A). At 75 days of cold storage, AG/Ch + 4% Zn–NPs (49.07) recorded the highest TAC activity followed by AG/Ch + 2% Zn–NPs (46.82) then AG/Ch (46.42) treatment, while control (33.33) recorded the lowest significant value. All Zn–NPs treatments increased TAC significantly compared to Cont. Also, mixing AG/Ch (46.42) gave a significantly higher value than individual Ch (44.97) or AG (44.67) at 75 days of cold storage.

CAT activity increased progressively with increasing periods of storage (Fig. 6B). At the end of storage period (75 days) Ch (12.50), AG(12.50), AG/Ch enriched Zn–NPs gave the highest significant CAT activity (12.50) compared to control (8.27). MDA decreased progressively with increasing storage periods (Fig. 6C). After 75 days of cold storage, AG/Ch (13.87) recorded the highest significant MDA activity followed by AG/Ch + 2%Zn–NPs (12.90) compared to the other treatments, while control (6.67) recorded the lowest significant value. All Zn–NPs treatments significantly increased MDA compared to Cont. Also, mixing AG/Ch (13.87) gave a higher significant value than individual Ch (10) or AG (11.91) during all cold storage periods.

POD activity increased gradually with increasing storage period (Fig. 6D). After 75 days of cold storage Ch (16.45) followed by both AG (15.61) and AG/Ch + 2 Zn–NPs (15.54) recorded the highest significant values while control (9.37) recorded the lowest one. All Zn–NPs treatments increased POD significantly compared to Cont.

PPO activity gradually increased with increasing storage periods (Fig. 6E). At 75 days of cold storage, the lowest PPO value (12.04) was recorded by AG/Ch + 4% Zn–NPs followed by AG/Ch (12.78), while control (21.93) recorded the highest significant value. Mixing of AG/Ch (11.63, 12.77) significantly reduced PPO at 60 and 75 days of cold storage compared to individual use of Ch (12.63, 16.78) and GA (13.13, 16.28). Also, Zn–NPs treatments significantly decreased PPO activity compared to Cont.

These results were consistent with Tesfay et al.^77^ as they found activation of physio-biochemical processes during storage of avocado fruit results in changes in peroxidase and catalase activity. Switala and Loewen^78^ found that CAT is more effective than POD in the degradation of H_2_O_2_. Coating strawberries with 15% GA significantly reduced the activity of polyphenol oxidase (PPO) activity, while it increased antioxidant capacity^50^. Also, Arabic gum (10%) + 1000 ppm *Moringa oleifera* seed oil treatment decreased the activity of polyphenol oxidase^33^. Moreover, Rashedy et al.^62^ found that turmeric acid recorded the lowest significant PPO activity while the control treatment recorded the highest PPO activity after the end of cold storage.

Mahmoudi et al.^79^ found that plum fruit coated with chitosan/Arg NPs (0.5%) enhanced activity of antioxidant enzymes during. Furthermore, Li et al.^80^ reported that nano-ZnO-enriched packaging film for preserving apple fresh-cut reduced MDA and inhibited POD and PPO. Moreover, chitosan/Nano-Titanium coating increased the activity of PPO, POD, while reducing MDA content^63^. Gray pulp disorder occurs in cold-stored fruits during ripening is associated with fruit senescence and Polyphenol oxidase (PPO) enzyme activity^75^. Also, Ch applications promoted the highest total antioxidant activity of Red Raspberries Piccolo^14^.

The results indicated that during the storage period there was a significant increase in CAT, POD, PPO activities as well as a significant decrease in MAD. Conversely, TAC increased till 45 days of storage and then decreased. Regarding the effect of treatments, it can be concluded that, Zn–NPs, Ch and AG treatments recorded high activity of TAC, CAT, MDA and POD with lower activity of PPO at the end of storage period (75 days) compared to the control. Individual application of Ch and AG slightly higher significant effect in CAT, MDA and POD followed by AG/Ch + 2% Zn–NPs. While AG/Ch + Zn–NPs at 8% recorded the highest TAC and the lowest PPO than individual application of Ch, AG as well as control treatments. These results were consistent with Pasquariello et al.^13^ who recorded enhancement of catalase activity and inhibition of polyphenol oxidase activity, which extends the shelf life of loquat fruit treated with Ch.

Our results indicate that AG/Ch + Zn–NPs treatments significantly reduced fruit decay percentage and maintained higher fruit firmness and good color during cold storage (Fig. 1a, b), which may be attributed to the antifungal properties of Zn–NPs^25–30^ as well as Ch^10,11^ attributed to lower reactive oxygen species accumulation and higher TAC as well as the higher activities of POD and CAT enzymes with lower activity of PPO activity. Our results were in agreement with^81^ who reported that, melatonin-treated fruit exhibited higher TAC due to lower H2O2 radicals and higher activities of antioxidant enzymes (CAT, POD, and MDA)in blueberries. The reduction in fruit decay may be contributed to higher CAT and POD activities in Zn–NPs, Ch and AG-treated avocado fruit.

## Conclusion

Individual application of Ch and AG succeed in maintaining fruit quality of Hass fruit during cold storage. Also, the combined application of Ch/AG was more effective than individual application of Ch or GA in decreasing fruit weight and PPO as well as increasing TAC. Moreover, enriched Ch/GA with 8% Zn–NPs recorded the lowest fruit weight loss, decay percentage, TSS content, and improved fruit skin and pulp color significantly compared to combined application of Ch/GA as well as the control treatment. Furthermore, Ch/AG + 8% Zn–NPs was the most recommended treatment which recorded the highest TAC and the lowest PPO than the combined application of Ch/GA as well as the control treatment. Moreover, enriched Ch/GA with Zn–NPs recorded the highest CAT and POD compared to the control.

## Data Availability

The datasets generated during and/or analyzed during the current study are available from the corresponding author on reasonable request.

## Abbreviations

AG: Arabic gum
ANOVA: Analysis of variance
CAT: Catalase
Ch: Chitosan
DSC: Differential scanning calorimetry analysis
FTIR: Fourier-transform infrared
MDA: Malondialdehyde
POD: Peroxidase
PPO: Polyphenol oxidase
CRBD: Complete randomized block design
SE: Standard error
SEM: Scanning electron microscopy
TAC: Total antioxidant capacity
TEM: Transmission electron microscope
TGA: Thermogravimetric analysis
WVT: Water vapour transmission rate
Zn–NPs: ZnO-nano particles
